# Evaluation of sampling strategies for assessing lymphatic filariasis endemic status of a non-MDA district in South India

**DOI:** 10.1371/journal.pntd.0013192

**Published:** 2025-06-25

**Authors:** Adinarayanan Srividya, Rajendran Dhanalakshmi, Raja Jeyapal Dinesh, Palappurath Maliyakkal Azad, Ramalingam Balasubramaniyan, Sivaprakasam T. Selvavinayagam, Palani Sampath, Masilamani Senthilkumar, Dhandapani Bharani Kumar, Brindha Balan, Philip Raj Abraham, Ashwani Kumar, Swaminathan Subramanian, Manju Rahi

**Affiliations:** 1 ICMR-Vector Control Research Centre, Gorimedu, Puducherry, India; 2 Department of Public Health and Preventive Medicine, Chennai, Tamil Nadu, India; Zhejiang Wanli University, CHINA

## Abstract

**Background:**

India is moving towards the Lymphatic Filariasis (LF) elimination goal in 2027. Documentation on LF transmission status in the non-endemic and unsurveyed areas is crucial for WHO to certify that LF has been eliminated as a public health problem in the country. Appropriate sampling strategy is necessary to determine LF transmission status in the areas not under mass drug administration (MDA). We evaluated four different sampling strategies to identify the best tool(s) and indicator(s) that could be used to assess transmission interruption in a non-MDA district.

**Methodology:**

This study was conducted in Salem district in Tamil Nadu, India, during the period from June 2022 to June 2023. Four different sampling strategies, namely: (i) School based Mini-TAS (Mini-sTAS, n = 480), (ii) Community based Mini-TAS (Mini-cTAS, n = 480), (iii) Molecular xenomonitoring surveys (MX, n = 7500), and (iv) Purposive sampling of five high-risk sites (human, n = 1500 and vector surveys, n = 3750), were evaluated for their ability to assess LF transmission status in the area. These strategies were compared with a large-scale community survey (n = 10200) in 30 randomly selected sites (villages/wards) assessing human infection in the study area. While Filariasis Test strips (FTS) were used to assess circulating filarial antigen (CFA), night blood smears from CFA positives were collected to assess microfilaraemia (Mf). Mosquito samples collected from MX surveys were subjected to polymerase chain reaction (PCR) assays to assess the infection in vectors.

**Results:**

The results of the large-scale survey showed that the overall prevalence of CFA was 0.2% (95% CI: 0.1%–0.3%), below the critical threshold of 2%. Mini-sTAS and Mini-cTAS both showed that the CFA prevalence among children was below the elimination threshold of 2%. MX surveys showed the vector infection prevalence of 0.03% (95% CI: 0.01%–0.09%). These three strategies showed that the district is non-endemic and corroborate the results of a large-scale community survey. However, under the purposive sampling strategy, in two high-risk sites, either human or vector infection prevalence was above the respective elimination thresholds. Further, the administrative blocks in which these sites were situated shared borders with known LF endemic districts.

**Conclusions:**

The sampling strategies that may be recommended for a non-MDA or unsurveyed district to assess LF transmission status would be to use (i) school- or community-based Mini-TAS or (ii) conduct MX surveys to classify them as endemic or non-endemic based on the pre-defined thresholds by WHO. For further confirmation, serosurveys among adults may be conducted in five purposively selected high-risk sites to identify pockets of LF transmission, if any.

## Author summary

Lymphatic filariasis is a mosquito-borne parasitic disease caused by filarial worms. India’s successful implementation of the elimination strategy in all 345 endemic districts has resulted in stopping MDA in 138 districts, and 68 have completed all three serial TAS. As more districts stop MDA, India will move towards certification, and the process requires showing that even the non-endemic or unsurveyed areas are free of LF. We have evaluated the existing sampling strategies used in endemic areas for assessing transmission and found that both school- and community- based confirmatory mapping tools (Mini-sTAS, and Mini-cTAS) and the molecular xenomonitoring for vector infection were sensitive strategies to assess LF endemicity in non-MDA or unsurveyed areas.

## Introduction

Globally, lymphatic filariasis (LF) remains a significant public health concern, necessitating considerable efforts for its elimination. The World Health Organization (WHO) recommends mass drug administration (MDA) with antifilarial drugs as the primary strategy for LF elimination. Accounting for 55% of the global LF burden, India is currently marching towards the revised LF elimination goal by 2027 [1]. India launched the MDA programme with diethylcarbamazine plus albendazole (DA) in all the 256 endemic districts in 2004, and over the years, as new districts were identified as endemic, the programme was expanded to 345 by 2024 [2]. The programme has made significant progress as 138 districts have stopped MDA after passing the WHO-recommended transmission assessment survey (TAS) and are under post-MDA surveillance. Following WHO recommendation [3], India implemented MDA with a triple drug regimen (ivermectin, diethylcarbamazine, and albendazole [IDA]) initially in five endemic districts on a pilot scale, and it was extended to 58 more endemic districts to accelerate LF elimination in the country. More districts are expected to move towards post-MDA surveillance after stopping MDA, as reported by the National Center for Vector Borne Diseases Control (NCVBDC), Delhi [4].

While launching the LF elimination programme in 2004, all the districts historically known to be endemic were included. However, there are districts in India whose LF endemicity status is still not known or uncertain. A geo-environmental risk model had predicted low to medium LF risk in some of these districts which were not under MDA [5]. Additionally, reports from Madhya Pradesh and among migrant populations in Batinda, Punjab, have indicated the presence of microfilaria (Mf) in districts currently not under MDA. The lack of countrywide mapping protocol for assessing LF endemicity prior to the introduction of the LF elimination programme underscores the necessity to assess LF status in districts assumed to be non-endemic or not under MDA to fulfil the validation criteria for certification of elimination by the WHO [6].

In the recent past, the National Vector Borne Diseases Control Programme (NVBDCP) recommended remapping of non-MDA or unsurveyed districts using Mini-TAS (the confirmatory mapping tool recommended by the WHO) [6–8], which measures the circulating filarial antigen (CFA) levels in 9–14 year-old children. However, studies that compared methods used for assessing infection in humans and vectors have shown that MX is more sensitive than CFA [9–11] in areas with low infection levels. MX detects parasite DNA in mosquitoes using the polymerase chain reaction (PCR) and indicates potential human infections [12,13]. While these tools have proven effective in assessing transmission interruption in endemic districts [14], their comparative evaluation in non-MDA areas is not documented.

This study aimed to develop strategies to assess LF endemicity in non-MDA or unsurveyed districts. We evaluated four different sampling strategies, namely (1) Mini-sTAS, (2) Mini-cTAS to assess infection in humans, (3) MX for vector infection and (4) Purposive sampling of high LF-risk communities to assess infection in both humans (circulating filarial antigen-CFA) and vectors (*W. bancrofti* parasite DNA prevalence). These methods were compared with (5) a large-scale community survey to ascertain their capacity to identify ongoing LF transmission and their implications in LF elimination. In addition, the study also explored the possible risk factors associated with filarial infection.

## Methods

### Ethics statement

This study was approved by the institutional ethics committee (ICMR-Vector Control Research Centre Institutional Human Ethics Committee approval certificate no. IHEC/0121/N/A dated 27/4/2021). Necessary approvals/permissions to conduct the study in the Salem district were obtained from the Tamil Nadu state and Salem district health and education authorities.

Parent or Legally Authorized Representative and School Principal written consent was obtained for children aged 5–17 years, and verbal/written assent, as applicable, was obtained for children aged 7–17 years. Written informed consent was obtained for all consenting adults aged 18 years and above.

### Study area

The study was conducted in the Salem district of the Tamil Nadu state in South India that has been listed as not endemic for LF [2].

**Fig 1:**
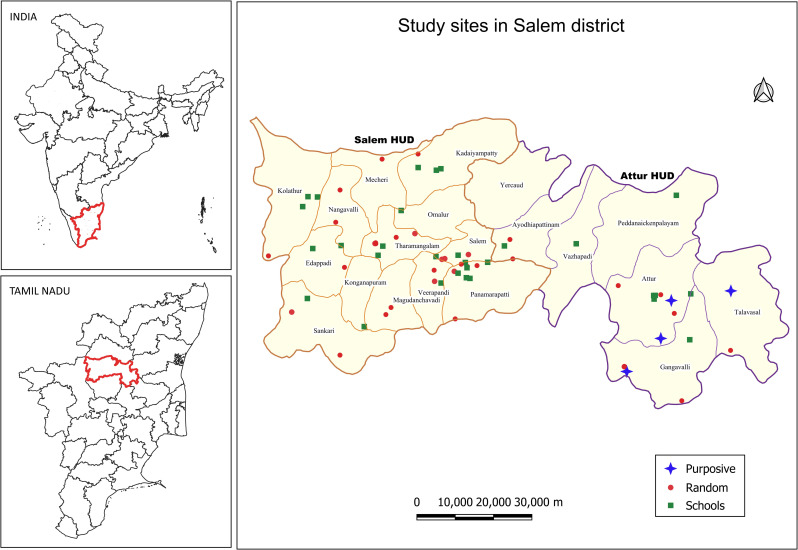
Study sites (Random and purposively selected villages/wards or randomly selected schools) in the Salem district with two Health Unit Districts (HUD): Salem and Attur. Base layer of map: https://onlinemaps.surveyofindia.gov.in. License information: https://onlinemaps.surveyofindia.gov.in/GeospatialGuidelines.aspx. ArcGIS Software: https://www.arcgis.com.

The Salem district, with a population of 3,482,000 comprising 20 blocks, is situated between 11°14’ and 12°53’ in North Latitude and between 77°44’ and 78°50’ in East longitude and is bounded on the North by Dharmapuri District, South by Trichy and Namakkal District, East by Villupuram and Perambalur Districts, and West by Erode District, of which Perambalur, Trichy and Villupuram are endemic for bancroftian filariasis. This district recorded the highest number of line-listed LF disease cases (144) as per the list obtained from the Tamil Nadu State NCVBDC, and therefore was assessed for the presence of transmission of bancroftian filariasis. The line listed cases within the Salem district were obtained from the office of Deputy Director of Health Services, Salem, which consolidates the data from all the Primary Health Centres (PHCs) of the district. The Salem district is divided into two Health Unit Districts (HUDs) - Salem and Attur (Fig 1) due to its vastness and population. The district has 87 PHCs and 461 Health Sub-centres (HSCs) situated in 18 administrative blocks (each block with an approximate population of 0.5 million).

### Sampling strategies

**Fig 2:**
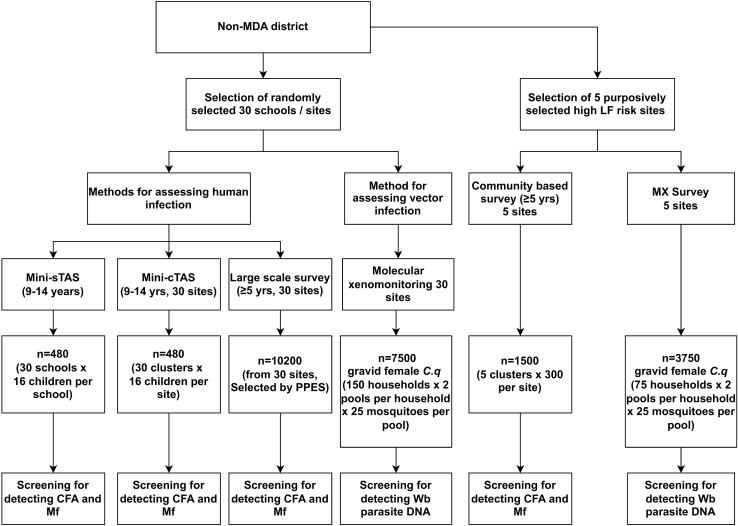
Study design and sample sizes. CFA- circulating filarial antigen, *C.q– Culex quinquefasciatus*, LF- lymphatic filariasis, Mf- Microfilaria, MX- Molecular xenomonitoring, PPES- Probability proportional to estimated size, *Wb- W. bancrofti*.

As indicated earlier, the following four different sampling strategies were carried out: school-based Mini-TAS (Mini-sTAS), community-based Mini-TAS (Mini-cTAS), MX for vector infection, and purposive sampling of high-risk sites (for both human and vector infection) were evaluated for their capacity to assess LF endemicity in the Salem district and were compared with a large scale survey (Fig 2). The details of the strategies are explained in detail below.

i. Mini-sTAS: As per the programme guidelines, the WHO recommended school based two-stage cluster sampling protocol for confirmatory mapping (Mini-TAS) was used for this strategy. Under this, the Survey Sample Builder Ver. 1.6 tool [15] was used to select 30 schools from all over the district based on the enrollment rate. The total number of children in the target age group (9–14 years) present on the day of the survey were noted from the attendance registers, and children were selected systematically in each school and tested for the presence of CFA and Mf among CFA positives. (Fig 2).
ii. Mini-cTAS: Thirty HSCs were first selected based on probability proportional to estimated population size (PPES), and samples were allocated proportionally to each HSC. To achieve this sample size, one village/ward was randomly selected from each HSC (Table A in S1 Text). Accordingly, a total of 23 villages and 7 wards were selected, and a minimum of 16 children aged 9–14 years were tested for CFA from the systematically selected households (HHs) in these communities. For this, we obtained the total number of HHs and children in that age group in each selected site from the Anganwadi family register. Based on the total households, we calculated the number of HHs that need to be surveyed to get those 16 children.
iii. MX: The sampling protocol developed and validated at EU levels by Subramanian et al., 2020 [14] (150 HHs x 2 pools x 25 gravid female *Culex quinquefasciatus* per pool) was used in the same 30 sites as that used in the Mini-cTAS. The number of houses to be sampled from each site was in proportion to the total number of houses in those clusters. The HHs were systematically selected for placing traps, and these were not the same HHs surveyed under Mini-cTAS.
iv. Purposive sampling: Five high-risk sites with the highest number of chronic LF cases (from the record of line-listed LF chronic cases (morbidity data) obtained from the district NCVBDC office) were selected. These sites were not part of the 30 sites selected for Mini-cTAS/MX surveys. As these sites are known to have the risk of LF transmission [16], 300 individuals aged 5 years and above from each site were tested for the presence of CFA. Based on the results of the human surveys, it was decided to carry out MX surveys in these sites to see if it can capture any signals of transmission. However, due to time constraints, it was targeted to sample at least 50% of the required sample size of 7500. Accordingly, we collected 3750 gravid female *C. quinquefasciatus* (75 HHs x 2 pools x 25 *mosquitoes per pool*) from all five sites.
v. In addition, a large-scale, community based human blood survey among individuals aged 5 years and above with a minimum sample size of 10,200 (assuming an expected Mf prevalence of 1%, precision of 0.25%, 95% CI, design effect of 1.5 and a non-response of 10%) was also carried out in the same 30 sites selected for Mini-cTAS/MX surveys.

### Blood surveys

A unique barcode was assigned to every consenting participant. The participants’ demographic details were collected in a proforma, and simultaneously real-time data entry was done using an in-house- developed mobile app in the field. A finger prick blood sample (75 µl) was collected for detecting CFA using the Filariasis Test Strip (FTS, Abbott) [17]. The FTS testing was done according to the manufacturer’s instructions, and the results were read at 10 minutes and noted in the proforma/app. The positive tests were scored based on the colour intensity of the test line in comparison to the control line as follows: 1+ Mild positive (test line colour less than that of control line); 2+ Moderate positive (colour intensity of test line same as that of control line); and 3+ Strong positive (colour intensity of test line more than that of control line).

Night blood smears were collected only from those who were CFA positive. Approximately 60 µl of finger prick blood was collected using a capillary tube, and three parallel lines of approximately 20 µl (smears) each were made on the slide, as per WHO guidelines [18]. The slides were air-dried, packed and later stained with Giemsa following standard operating protocol. The stained smears were examined in the laboratory under a microscope for the presence of microfilaria.

### Vector surveys

In the MX survey, the hay infusion was prepared with 200 g of hay soaked in 45 litres of tap water along with 2 g each of yeast and malt for 5–7 days. By then, the water ferments and becomes a suitable attractant for gravid female mosquitoes. Modified CDC gravid traps [19] (with the above described hay infusion) were placed in the selected households to attract the female gravid *C. quinquefasciatus* mosquitoes after getting verbal permission from the head of the household. Traps were placed outdoors in a suitable place (not disturbed by movement of people and in a dark place) to collect mosquitoes for a maximum of three days. The traps were placed in the evenings before 6 pm and were collected in the morning by a team before 8 am. The mosquitoes brought to the lab were segregated, and the female *C. quinquefasciatus* mosquitoes (fully fed, semi-gravid, and gravid) were equally distributed in two vials each, labelled with unique barcodes as A and B vials. This was done so as to obtain consistent results. Every day’s collection was equally distributed in these two vials, and these were placed in the dry bath for 24 hours to prevent fungal growth. The procedure was repeated for three days of collection or until both the vials had 25 gravid female *C. quinquefasciatus* mosquitoes, whichever is earlier. During vector surveys, a proforma was also used to collect the details of the areas surveyed, geo-coordinates of the household, the number of mosquitoes collected in each pool, their gravid status, etc.

### PCR Assays to determine vector infection

The mosquito samples were brought to the headquarters and were processed in the lab through PCR assays [20,21] to detect the presence of the *W. bancrofti* DNA in the pools of samples [13].

Filarial parasite DNA was extracted from each mosquito pool following an indigenously developed simple TE (Tris-EDTA) based DNA extraction procedure using bead beating (BB) for grinding the mosquitoes [22]. The DNA samples thus obtained were coded and analyzed by real-time quantitative PCR as described elsewhere [20,21]. Briefly, each real-time PCR reaction was performed with 12.5 µl of FastStart Essential DNA probes Master (Roche Diagnostics, Germany) along with 450 nmol/L of each primer: LDR1–5’ATTTT GATCATCTGGGAACGTTAATA-3’; LDR2–5’CGACTGTCTAATCCATTCAGAGTGA-3’ and 125 nmol/L probe (6 FAM-ATCTGCCCATAGAAATAACTACGGTGGATCTG-TAMRA) (IDT, USA) in a final volume of 25 µl in 96-well MicroAmp optical plates (Roche Diagnostics, Germany). One microlitre of the extracted DNA was used as a template in each real-time PCR as described earlier, along with 1 ng, 100 pg and 10 pg of purified genomic DNA samples as positive controls and water controls. Technicians trained in real-time PCR assays were involved, and all the reactions were run in duplicates. The cycle of quantification values for each sample was in terms of a single value reflecting the cycle number needed for quantification. Thermal cycling parameters were 50^o^C for 2 minute, 95^o^C for 10 minute followed by 40 cycles of 95^o^C for 15 seconds and 60^o^C for 1 minute. Thermal cycling and data analysis were done with the Light Cycler 96 (Roche, Germany) instrument using sequence detection system (SDS) software (Applied Biosystems). The cycle of quantification values of samples ranging from 1.0–39.0 were considered positive, and samples that failed to reach the fluorescence threshold beyond 39 were considered indeterminate and repeated to confirm the negativity or positivity of those samples as described elsewhere [23].

### Statistical analysis

The results of the sampling strategies were assessed as follows:

Under Mini-TAS (school or community-based), if the number of CFA positives ≥ 4, the district was considered as ‘endemic’ [7,8].

In community/large-scale surveys, if the CFA prevalence was ≥ 2% or the Mf prevalence was ≥ 1% it indicated ongoing transmission and considered ‘endemic’.

For MX surveys, PoolTestR was used to estimate the vector infection rate [24]. If the estimated vector infection rate is above the WHO-recommended provisional threshold of ≥0.25%, the district was considered as ‘endemic’ [25].

The results of different strategies—Mini-sTAS, Mini-cTAS, purposive sites survey, and MX—were compared with those obtained from large-scale community survey to evaluate the appropriateness of each sampling strategy to assess the LF transmission status.

The maps with the study sites and sites positive for filarial infection were produced using the software ArcGIS version 10.2 [26]. The shape-files were downloaded from the Survey of India website with the license information.

Multivariate logistic regression analysis was carried out to find the factors (age, gender, vector infection status of the community they reside in, and if the block where the community they reside in shares borders with an endemic district) associated with the CFA status of an individual. The analysis was carried out separately for random and purposive sites. For assessing the significance of the model and predictors, a p-value of <0.05 was considered significant. All the analyses were done using the Stata Software V.14.0 [27].

## Results

### LF situation at the district level

#### Large scale community survey (30 random sites).

A total of 10388 individuals were screened for CFA, of which 6345 (61%) were females and the remaining were males. A total of 18 CFA positives were detected from 12 sites, of which 9 (0.14%, 95% CI: 0.09 – 0.23%) were females and rest males (0.22%, 95% CI: 0.15–0.37%). All CFA positives except three were aged above 18 years, with a mean age of 41 years (Range 6–75 years). All these positives were natives of the Salem district, residing for more than six years with no history of visiting/residing in any endemic districts in the past. Of these 18 CFA positives, only four (including one child aged six years) tested moderately positive (2+), and the rest were mild positive (1+). None of the CFA positives were found to be Mf positive.

**Fig 3:**
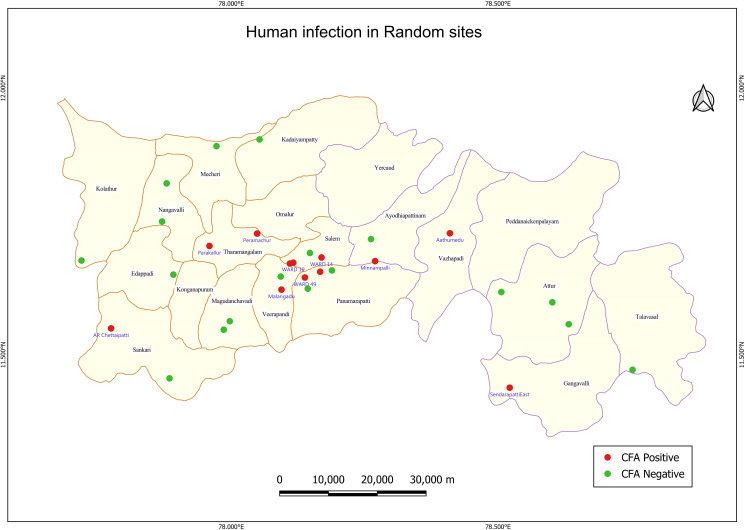
Sites with circulating filarial antigen (CFA) positives in the 30 random sites of Salem district. Base layer of map: https://onlinemaps.surveyofindia.gov.in. License information: https://onlinemaps.surveyofindia.gov.in/GeospatialGuidelines.aspx. ArcGIS Software: https://www.arcgis.com. Legend: CFA- circulating filarial antigen.

**Fig 4:**
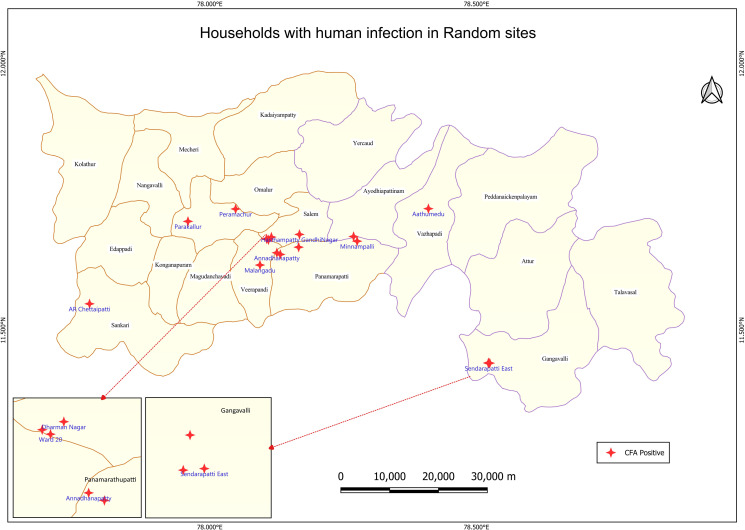
Households with circulating filarial antigen positives in 30 random sites, Salem district. Base layer of map: https://onlinemaps.surveyofindia.gov.in. License information: https://onlinemaps.surveyofindia.gov.in/GeospatialGuidelines.aspx. ArcGIS Software: https://www.arcgis.com. Legend: CFA- circulating filarial antigen.

The CFA prevalence across the sites ranged between 0.1% and 2.8%, with only one site having CFA prevalence >2%. (Table A in S1 Text). Spatial distribution of CFA positive sites (even if there is one CFA positive) is shown in Fig 3. It may be seen that clustering of households with CFA positives was observed in a few sites (Fig 4).

### Sampling strategies tested for assessing filarial endemicity

**Fig 5:**
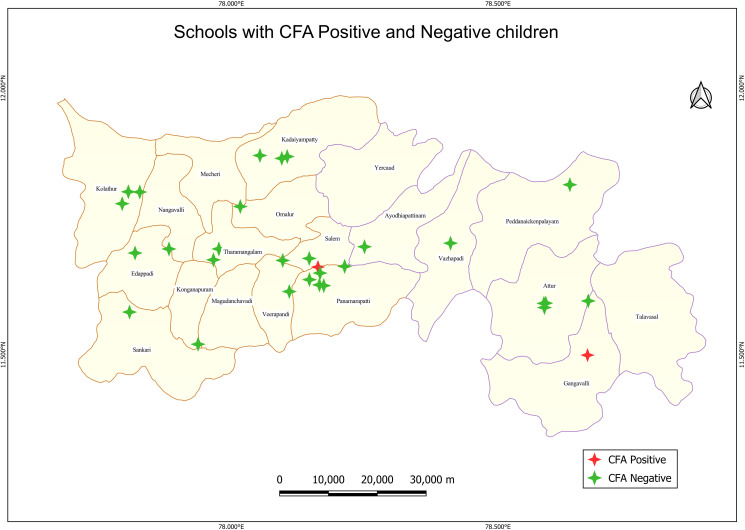
Distribution of schools with circulating filarial antigen (CFA) positive children in Salem district. Base layer of map: https://onlinemaps.surveyofindia.gov.in. License information: https://onlinemaps.surveyofindia.gov.in/GeospatialGuidelines.aspx. ArcGIS Software: https://www.arcgis.com. Legend: CFA- circulating filarial antigen.

1. Mini-sTAS (30 schools):

A total of 479 children aged 9–14 years from 30 schools across 19 blocks were screened for filarial infection, of which 3 tested positive for CFA (Table B in S1 Text). One child was from Salem HUD, and two were from Attur HUD and these children were natives of the Salem district. The sampled schools and those with CFA positives are shown in Fig 5.

1. Mini-cTAS:

A total of 489 children aged 9–14 years were selected randomly from the 30 random sites (16 per community) and tested for the presence of CFA. However, none of them tested positive.

1. Vector infection using MX:

A total of 10,531 mosquito species were collected using the gravid traps, of which *C. quinquefasciatus* constituted 83.4%, *Aedes* 0.1%, *Armigeres* 3.3% and the rest (*Anopheles, Mansonioides, etc.*) 13.2%.

**Fig 6:**
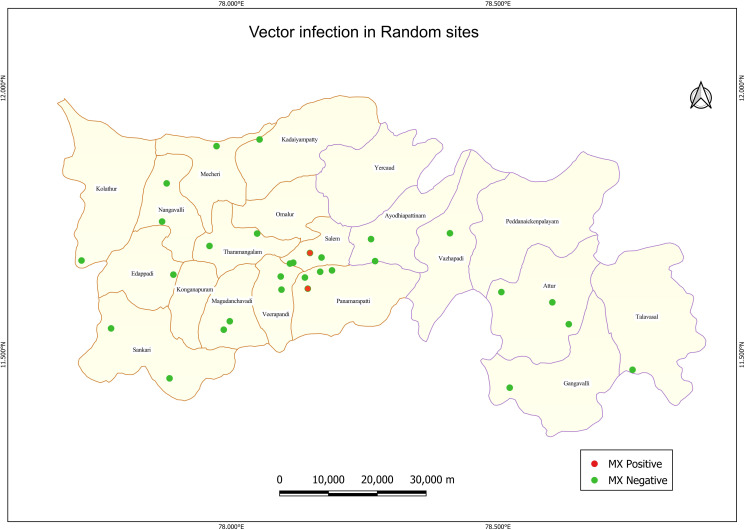
Distribution of sites with vector infection in the 30 random sites of Salem district. Base layer of map: https://onlinemaps.surveyofindia.gov.in. License information: https://onlinemaps.surveyofindia.gov.in/GeospatialGuidelines.aspx. ArcGIS Software: https://www.arcgis.com. Legend: MX- Molecular xenomonitoring.

A total of 6898 female *C. quinquefasciatus* mosquitoes (fully fed, semi-gravid and gravid) were collected in two vials each from 152 systematically selected households from the 30 random sites. A total of 304 pools were collected, of which only 2 sites (Ward 5 and Ward 60A) recorded positive pools with a *W. bancrofti* parasite DNA prevalence 0.24% (95% CI: 0.01–1.12%) and 0.30% (95% CI: 0.02–1.40%) respectively (Table C in S1 Text). The overall *W. bancrofti* parasite DNA prevalence was found to be 0.03% (95% CI: 0.01–0.09%). The sites with vector infection in the 30 random sites are shown in Fig 6.

1. Purposive sites:

**Human infection**

A total of 1544 individuals aged 5 years and above were tested from the five high risk (purposively selected) sites. The site-wise details of filarial infection are shown in Table 1. Out of 1544 individuals, 18 (1.2%) tested positive for CFA. The upper 95% CI for CFA prevalence exceeded the 2% threshold for two sites, indicating ongoing transmission. None of these CFA positives were Mf positive.

**Table 1:** Site-wise circulating filarial antigen (CFA) positivity in the purposive sites of Salem district.

Name of the purposive site	No. examined	% CFA positive (95% CI)	Age or age-range (in years) of the CFA positives
Kadambur	301	0.3 (0.0 – 1.0)	62
**Navakurichi**	**305**	**2.6 (0.8 – 4.4)**	**30–78**
Pethanayakanpalayam	308	1.6 (0.2 – 3.0)	23–78
Sendarapatti	314	0.3 (0.0 – 0.5)	33
Thulakanur	316	0.6 (0.0 – 1.5)	24 & 54
Total	1544	1.1 (0.6 – 1.6)	23–78

In parenthesis: 95% CI - 95% confidence interval.

**Vector infection**

**Fig 7:**
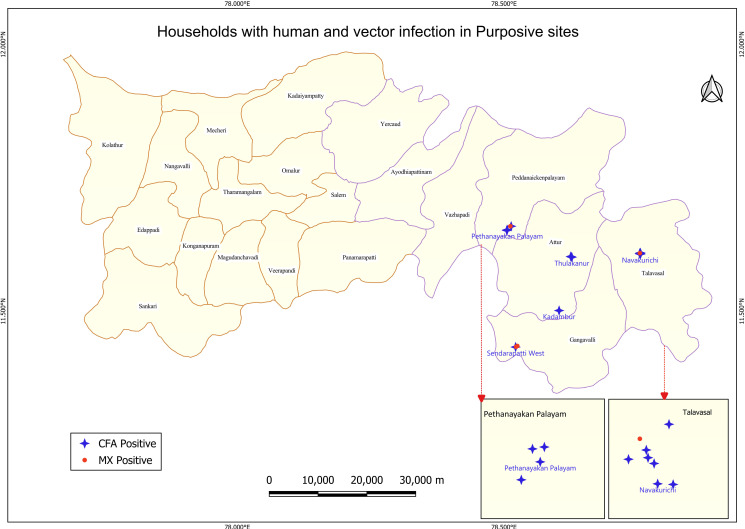
Distribution of households with human and vector infection in the 5 purposive sites. Base layer of map: https://onlinemaps.surveyofindia.gov.in. License information: https://onlinemaps.surveyofindia.gov.in/GeospatialGuidelines.aspx. ArcGIS Software: https://www.arcgis.com. Legend: CFA- circulating filarial antigen, MX- Molecular xenomonitoring.

A total of 150 pools with 4012 gravid female *C. quinquefasciatus* mosquitoes were collected from 75 households (15 households from each site), and it was observed that the upper 95% CI limit of all five sites exceeded the provisional LF elimination threshold of 0.25%, indicating the risk of transmission (Table 2). The HHs with human and vector infection in the purposive sites are shown in Fig 7.

**Table 2:** Site-wise vector infection prevalence in the purposive sites.

Name of the purposive site	No. of mosquitoes collected	No. of pools	No. of pools positive	*W. bancrofti* parasite DNA prevalence (%) (95% CI)
Kadambur	854	30	0	0.00 (0.00 – 0.59)
Navakurichi	708	30	1	0.19 (0.01 – 0.91)
Pethanayakanpalayam	813	30	1	0.17 (0.01 – 0.81)
**Sendarapatti**	**820**	**30**	**2**	**0.34 (0.06 – 1.09)**
Thulakanur	817	30	0	0.00 (0.00 – 0.62)

In parenthesis: 95% CI - 95% confidence interval.

### Comparison with large scale survey

While the CFA prevalence based on the large-scale survey was 0.2% (95% CI: 0.1–0.3%), the *W. bancrofti* parasite DNA prevalence based on the MX survey was 0.03% (95% CI: 0.01–0.09%). The results of the Mini-TAS, both community and school based, also indicate that the district is non-endemic, with nil CFA positives among children aged 9–14 years in the Mini-cTAS and only three positives in the Mini-sTAS. All three sampling strategies, namely Mini-cTAS, Mini-sTAS and the MX, indicate that the Salem district is non-endemic.

**Fig 8:**
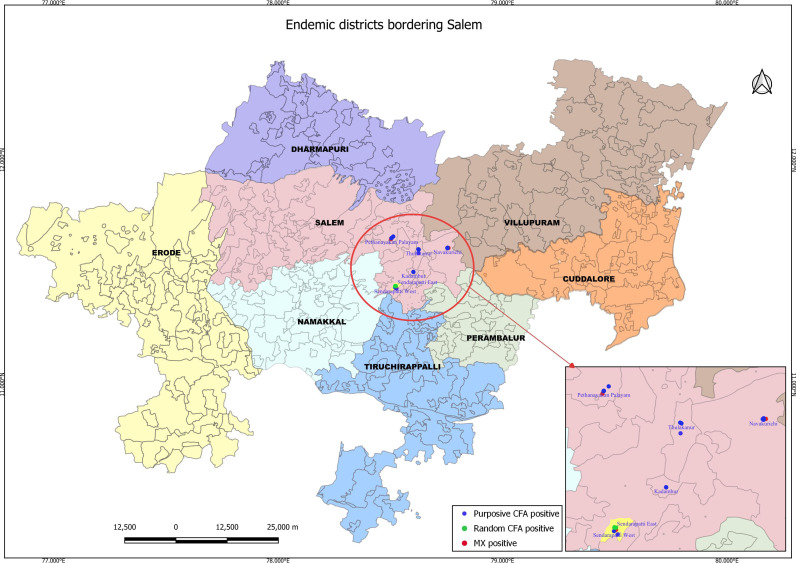
Administrative blocks with sites indicating signals of ongoing transmission, share borders with lymphatic filariasis endemic districts. Base layer of map: https://onlinemaps.surveyofindia.gov.in. License information: https://onlinemaps.surveyofindia.gov.in/GeospatialGuidelines.aspx. ArcGIS Software: https://www.arcgis.com. Legend: CFA- circulating filarial antigen, MX- Molecular xenomonitoring.

However, the purposive sampling strategy indicates ongoing transmission though at a low level, in the high-risk sites. It may be observed that all the five sites are situated in the administrative blocks that share borders with other LF endemic districts in Tamil Nadu (Villupuram, Trichy, Perambalur, and Cuddalore) (Fig 8).

### Factors associated with filarial infection (CFA)

#### Random sites.

The logistic regression analysis showed that the CFA positivity of an individual was significantly associated with residing in an administrative block that shares a border with a LF endemic district (χ^2^ = 14.3, p value = 0.013). The risk of being CFA positive was 5.4% (95% CI: 1.8–15.7%, p value = 0.002) times higher for an individual residing in a block that shares its administrative border with a LF endemic district compared to an individual residing in a block not sharing border with a LF endemic district. All other independent variables were not significantly associated with CFA positivity of the individual.

#### Purposive sites.

In the purposive sites, similar analysis showed that CFA positivity was associated with those above 40 years of age and those residing in an area with ongoing transmission by vectors. The CFA positivity in an individual was 3.0% (95% CI: 1.1–5.4%) times higher among those aged above 40 years compared to those aged <20 years and 4.4% (95% CI: 1.3–15.5%, p value = 0.021) times higher for those residing in a community positive for vector infection compared to those living in a community that did not have infected vectors. (χ^2^ = 17.7, p value = 0.001).

## Discussion

This study assesses different strategies to determine the LF transmission status in a non-MDA or non-endemic district in Tamil Nadu. We evaluated four different sampling strategies along with a large-scale community survey to assess the LF endemicity, providing important insights into their performance. Three out of the four strategies, namely Mini-sTAS, Mini-cTAS and MX in the randomly selected schools/sites, demonstrated that the district is non-endemic (≤3 CFA positives in Mini-TAS and <0.25% for the vector infection). The results of all these three methods were consistent with the results from a large-scale community survey, which indicated that the overall CFA prevalence (0.2%) in the district was much below the elimination threshold (2%). Based on the above results, it may be concluded that the district is non-endemic.

A recent study in two non-endemic districts from the Odisha State of India has recommended school-based Mini-TAS as the most accurate tool to decide on whether an area does or does not require MDA even when the infection levels are low, particularly among non-endemic or unsurveyed districts [28]. Mini-TAS confirmed the endemicity in one of the non-endemic districts, Balangir, that had recorded a high antigeneamia prevalence of 26.7% (95% CI: 20.8–32.5%) as early as 2009 [29] and hence has been found to be endemic even now, as it was not under MDA. In this study, Mini-TAS was conducted only among the schools in the 30 sites selected to conduct the community Mf survey, whereas in the current study the schools were selected all over the district as per the WHO guidelines of Mini-TAS [28], which had a better geographical coverage compared to the other study. As the results of the Mini-sTAS confirm the findings of the Mini-cTAS and MX in both studies, this tool can be used to assess the LF transmission status in non endemic districts.

However, the purposive sampling strategy, in which the surveys were conducted in the high-risk sites selected based on the number of LF diseased cases, provided evidence of ongoing LF transmission both in terms of human and vector infection, some of them exceeding the critical thresholds at site level. All the five sites recorded CFA positives, and three of them also recorded vector infection as well, indicating signals of ongoing transmission. These observations corroborate the findings of other studies [16,30,31] which recommended that use of morbidity data may be a practical proxy to trigger mapping of ongoing transmission in low endemic areas. In one of these studies [16], confirmatory mapping survey revealed ongoing transmission in two previously classified non-endemic districts. Further, it was observed that these districts shared boundaries with LF endemic districts similar to the findings in our study.

The non-MDA district Salem, where this study was conducted, is surrounded by LF endemic districts, namely Villupuram, Trichy, Perambalur, and Cuddalore on the southeastern side of the district. It may be noted that these endemic districts have successfully stopped MDA. However, there is always a risk of people with residual infection from these endemic districts travelling to non-MDA districts, resulting in transmission in these areas where there are no MDA/LF control programmes currently in place. Further, our study showed that an individual residing in an administrative block sharing a border with LF-endemic districts was significantly associated with CFA positivity, underscoring the impact of geographical proximity on transmission risk. Additionally, age and the presence of vector infection in the community were identified as significant factors in the purposive sites, providing insights into demographic and environmental determinants of LF transmission.

Yet another finding from this current study is that the MX could detect more precisely the ongoing LF transmission in the communities which the blood surveys could not. The MX survey showed ongoing transmission in three of the five purposive sites (Fig 7) with one of them recording 0.34% parasite DNA prevalence, above the provisional threshold of 0.25%. It was also noted that the upper 95% confidence limit was higher than the provisional threshold (0.25%) in all these high-risk sites (Table 2). Though the samples were not sufficiently powered to estimate the vector infection prevalence, still signals of transmission were captured effectively by MX, indicating the sensitivity of the tool as observed elsewhere. Further, clustering of the positive households for human and vector infection indicates the presence of ongoing transmission in Navakurichi, which also had a CFA prevalence of 2.6% (Fig 7). While the CFA positivity indicates the presence of adult worms (dead or alive) up to 1–5 years post treatment [32], MX positivity provides a signal of ongoing transmission [14,20], suggesting the circulation of Mf in the population. Detection of CFA and infection in vectors in these sites may indicate a new infection, particularly when there has been no MDA in these areas. Further, being in the administrative blocks that shared a border with LF endemic districts could have been a reason for this evidence of LF transmission, as has been seen elsewhere [16]. These findings suggest that surveillance may be required in these blocks to monitor for transmission.

Though costing of each of these strategies could not be done in this study, one of our earlier studies compared TAS (which again examines children aged 6–7 years in a school-based survey) and MX surveys and found that MX was marginally lower in cost [14]. In a recent study [28] in Odisha, India, that carried out similar strategies to identify districts that are endemic for LF, it was observed that the per capita cost under purposive sampling strategy was the least (Rs. 0.09), followed by MX (Rs. 0.15) and Mini-TAS (Rs. 0.19). The MX being a more sensitive tool, may be appropriate, but to implement this, there are costs involved in terms of infrastructure (PCR labs), reagents, manpower and finally, the expertise.

Assessing the non-MDA/unsurveyed districts for their LF transmission status is one of the important activities while moving towards certification by WHO for elimination of lymphatic filariasis as a public health problem. This study has identified strategies like Mini-TAS (school- or community-based) to be appropriate to rapidly assess LF transmission for the whole district, which also represents the district spatially.

The finding of this study suggests carrying out blood surveys (for detection of CFA in adults aged 20 years and above) and/or MX particularly in areas that share borders with endemic districts. This would ultimately help the health authorities to take appropriate measures to prevent the introduction of disease or the establishment of a transmission focus in the fringe areas bordering the endemic districts.

### Strengths and limitations

The study comprehensively assessed multiple sampling strategies confirming the district as non-endemic through consistent findings from three out of four strategies. This consistency reinforces the reliability of these methods in confirming the non-endemic status at a broader level. The large-scale community survey in 30 random sites contributed to the overall assessment of LF endemicity in the district and provided valuable data indicating CFA prevalence and parasite DNA prevalence were well below the LF elimination thresholds. The study identified ongoing LF transmission in high-risk areas, highlighting the necessity of targeted surveillance. However, our study had a few limitations, the first one being the underpowered site-level sample sizes estimating infection levels in vectors in the purposive sites, and therefore the results regarding parasite DNA prevalence need to be treated with caution. As for the second limitation, it was about not conducting cost analysis for various strategies which could have helped in assessing their economic feasibility.

## Conclusions

Evaluation of the four sampling strategies at the evaluation unit (district) level suggest that while school- or community-based Mini-TAS or MX surveys (if resources permit) may be effective in confirming the endemicity status at a broader level, targeted strategies like purposive sampling of high-risk areas (based on the data of line-listed LF disease cases) and testing adults aged 20 years and above for the presence of CFA or Mf can be used for identifying pockets of transmission, if any, in the district. If the CFA antigeneamia and/or vector infection levels based on Mini-TAS or MX surveys are above the provisional LF elimination threshold of 2% and/or 0.25%, respectively, for those blocks containing the positive sites, the district can either opt for a test and treat policy or MDA at the block level to clear the foci of transmission with further surveillance.

## Supporting information

S1 Text **Table A.** Details of large-scale community survey conducted in 30 randomly selected sites in Salem district. **Table B.** Details of Mini-sTAS conducted in 30 randomly selected schools in Salem district. **Table C**. Details of vector infection in terms of *W. bancrofti* parasite DNA prevalence in the 30 random sites in Salem district.
(DOCX)
