# Supplementary material for: Evaluation of sampling strategies for assessing lymphatic filariasis endemic status of a non-MDA district in South India
Source: PLoS Negl Trop Dis. 2025 Jun 25;19(6):e0013192. doi: 10.1371/journal.pntd.0013192 (PMC12192127; doi:10.1371/journal.pntd.0013192)
Supplement: S1 Text — Table A. Details of large-scale community survey conducted in 30 randomly selected sites in Salem district. Table B. Details of Mini-sTAS conducted in 30 randomly selected schools in Salem district. Table C. Details of vector infection in terms of W. bancrofti parasite DNA prevalence in the 30 random sites in Salem district. (DOCX) [file pntd.0013192.s001.docx]

**S1 Text**

Details of large-scale community survey conducted in 30 randomly selected sites in Salem district (Table A)

| **Table A : Site-wise CFA positivity in the 30 random sites in Salem District** | | | | | | |
| --- | --- | --- | --- | --- | --- | --- |
| **HUD** | **Block** | **Random sites** | **HSC population** | **Site population** | **No. examined** | **CFA positive (95% CI)** |
| Attur | Ariyapalayam | Olapaddi | 6886 | 1553 | 123 | 0.0 |
| Attur | Ayothipattinam | Sukkampatti | 4974 | 1675 | 90 | 0.0 |
| Attur | Belur | Aathumedu | 9339 | 3185 | 162 | 0.6 (0.0 -1.8) |
| **Attur** | **Gangavalli** | **Sendarapatti East** | **8348** | **2093** | **143** | **2.8 (0.1 -5.5)** |
| Attur | Karipatti | Dasanaickenpatti | 5040 | 968 | 89 | 0.0 |
| Attur | Karipatti | Minnampalli | 9410 | 4872 | 169 | 1.2 (0.0 -2.8) |
| Attur | Malliakarai | Manjini | 4644 | 2995 | 83 | 0.0 |
| Attur | Thalaivasal | Nallur | 7034 | 1106 | 131 | 0.0 |
| Salem | Kadaiyampatti | Jodukuligundakkalpudur | 7885 | 2782 | 136 | 0.0 |
| Salem | Konganapuram | Periyamuthaiyampatti | 6760 | 720 | 118 | 0.0 |
| Salem | Magudanchavadi | Pethanur | 12227 | 1656 | 216 | 0.0 |
| Salem | Mecheri | Vellar | 4976 | 707 | 86 | 0.0 |
| Salem | Mettur | Sathyanagar | 9944 | 1421 | 194 | 0.0 |
| Salem | Nangavalli | Kandanur North | 8296 | 312 | 155 | 0.0 |
| Salem | Nangavalli | Periyavanavasi | 8495 | 1134 | 149 | 0.0 |
| Salem | Omalur | Guruvareddiyur | 8314 | 869 | 145 | 0.0 |
| Salem | Omalur | Peramachur | 9553 | 1599 | 178 | 0.6 (0.0 -1.7) |
| Salem | Panamarthupatti | Malangadu | 6748 | 1511 | 123 | 0.8 (0.0 -2.4) |
| Salem | Salem Corporation | WARD 14 | 58462 | 3012 | 1027 | 0.1 (0.0 -0.3) |
| Salem | Salem Corporation | WARD 19 | 58036 | 2841 | 1024 | 0.1 (0.0 -0.3) |
| Salem | Salem Corporation | Ward 20 | 59321 | 3078 | 1044 | 0.2 (0.0 -0.5) |
| Salem | Salem Corporation | Ward 33 | 58219 | 3537 | 1018 | 0.1 (0.0 -0.3) |
| Salem | Salem Corporation | WARD 49 | 60205 | 2398 | 1061 | 0.2 (0.0 -0.5) |
| Salem | Salem Corporation | WARD 5 | 54844 | 3944 | 963 | 0.0 |
| Salem | Salem Corporation | WARD 60A | 62269 | 2449 | 1083 | 0.0 |
| Salem | Sankari | AR Chettipatti | 5870 | 728 | 103 | 1.0 (0.0 -2.9) |
| Salem | Sankari | Sanyasipattiagraharam | 5232 | 510 | 93 | 0.0 |
| Salem | Tharamangalam | Parakallur | 11538 | 728 | 204 | 0.5 (0.0 -1.5) |
| Salem | Veerapandy | Kalparapatti | 7507 | 1742 | 144 | 0.0 |
| Salem | Veerapandy | Salamarathuvattam | 7470 | 1851 | 134 | 0.0 |
| **Grand Total** | | |  |  | **10388** | 0.2 (0.1 -0.3) |

Abbreviations: CFA- Circulating filarial antigen; HSC- Health Sub Centre; HUD- Health Unit Districts; 95% CI - 95% confidence interval

Details of Mini-sTAS conducted in 30 randomly selected schools in Salem district (Table B)

| **Table B: School-wise CFA positives in the Salem District** | | | | |
| --- | --- | --- | --- | --- |
| **HUD** | **Block** | ***Selected School and area** | **No. examined** | **No. CFA positives** |
| Salem | Salem | School 1, Erumapalayaam | 16 | 0 |
| Salem | Valapaddi | School 2, Belur | 16 | 0 |
| Salem | Sankari | School 3, Chettipatti | 16 | 0 |
| Attur | Mecheri | School 4, Chinthamaniyur | 16 | 0 |
| Salem | Kadayampatti | School 5, Kanjanaickenpatti | 16 | 0 |
| Salem | Kadayampatti | School 6, Kadayampatti | 16 | 0 |
| Attur | Ayothipattinam | School 7, Valasaiyur | 16 | 0 |
| Salem | Epappadi | School 8, Adaiyur | 17 | 0 |
| Attur | Thalaivasal | School 9, Kattukottai | 16 | 0 |
| Salem | Tharamanalam | School 10, Thuttampatti | 15 | 0 |
| Attur | PN Palayam | School 11, Pethanaickenpalayam | 16 | 0 |
| Attur | Attur | School 12, Attur | 16 | 0 |
| Salem | Salem | School 13, Erumapalayam | 16 | 0 |
| Attur | PN Palayam | School 14, Nathampatu | 16 | 0 |
| Salem | Salem | School 15, Gugai | 16 | 0 |
| Salem | Salem | School 16, Fairland, Salem | 16 | 0 |
| Salem | Edapaadi | School 17, Reddiyoor | 16 | 0 |
| Salem | Omalur | School 18, Thonnapalli | 16 | 0 |
| Salem | Veerapandy | School 19, Veerapandi | 16 | 0 |
| Salem | Salem | School 20, Periyamottur | 16 | 0 |
| Attur | PN Palayam | School 21, Pethanaickenpalayam | 16 | 0 |
| Salem | Nangavalli | School 22, Kunjandiyur | 16 | 0 |
| **Salem** | **Salem** | **School 23, Salem Rural** | **16** | **1** |
| Salem | Tharamanalam | School 24, Tharamangalam | 16 | 0 |
| Salem | Salem | School 25, Kitchipalayam | 15 | 0 |
| Salem | Magudanchavadi | School 26, Magudanchavadi | 16 | 0 |
| **Attur** | **Gangavalli** | **School 27, Gangavalli** | **16** | **2** |
| Salem | Panamarathupatti | School 28, Neikarapatti | 16 | 0 |
| Salem | Kolathur | School 29, Kolathur | 16 | 0 |
| Salem | Mettur | School 30, Mettur | 16 | 0 |
| **Grand Total** | | | **479** | **3** |

Abbreviations: CFA- Circulating filarial antigen; HUD- Health Unit Districts

*School names are anonymized to maintain confidentiality

Details of Vector infection in terms of *W. bancrofti* parasite DNA prevalence in the 30 random sites of the Salem district are shown in Table C

| **Table C: Site-wise *W. bancrofti*** **parasite DNA prevalence in random sites** | | | | | | |
| --- | --- | --- | --- | --- | --- | --- |
| HUD | Block | **Site name** | No. of female gravid *C.q*  mosquitoes | No. of pools | No. of positive pools | *W. bancrofti* parasite DNA prevalence (%)  (95% CI) |
| Attur | Ariyapalayam | Olapadi | 175 | 8 | 0 | 0.00 (0.00 - 1.77) |
| Attur | Ayothipattinam | Sukkampatti | 92 | 4 | 0 | 0.00 (0.00 - 2.88) |
| Attur | Belur | Athumedu | 400 | 16 | 0 | 0.00 (0.00 - 0.86) |
| Attur | Gangavalli | Sendarapatti East | 250 | 10 | 0 | 0.00 (0.00 - 1.29) |
| Attur | Karipatti | Dasanaickenpatti | 100 | 4 | 0 | 0.00 (0.00 - 2.66) |
| Attur | Karipatti | Minnampalli | 610 | 26 | 0 | 0.00 (0.00 - 0.59) |
| Attur | Malliakarai | Manjini | 299 | 16 | 0 | 0.00 (0.00 - 1.14) |
| Attur | Thalaivasal | Nallur | 140 | 6 | 0 | 0.00 (0.00 - 2.09) |
| Salem | Kadaiyampatti | Jodukuli | 307 | 14 | 0 | 0.00 (0.00 - 1.10) |
| Salem | Konganapuram | Periya Muthaiyanpatti | 86 | 4 | 0 | 0.00 (0.00 - 3.06) |
| Salem | Magudanchavadi | Pethanur | 186 | 8 | 0 | 0.00 (0.00 - 1.67) |
| Salem | Mecheri | Vellar | 100 | 4 | 0 | 0.00 (0.00 - 2.66) |
| Salem | Mettur | Sathya nagar | 200 | 8 | 0 | 0.00 (0.00 - 1.56) |
| Salem | Nangavalli | Kandanur North | 50 | 2 | 0 | 0.00 (0.00 - 4.20) |
| Salem | Nangavalli | Periyavanavasi | 150 | 6 | 0 | 0.00 (0.00 - 1.96) |
| Salem | Omalur | Guruvarediyur | 78 | 4 | 0 | 0.00 (0.00 - 3.39) |
| Salem | Omalur | Peramachur | 147 | 8 | 0 | 0.00 (0.00 - 2.10) |
| Salem | Panamarthupatti | Malangadu | 187 | 8 | 0 | 0.00 (0.00 - 1.66) |
| Salem | Salem Corporation | WARD 14 | 305 | 16 | 0 | 0.00 (0.00 - 1.12) |
| Salem | Salem Corporation | WARD 19 | 326 | 14 | 0 | 0.00 (0.00 - 1.03) |
| Salem | Salem Corporation | WARD 20 | 324 | 16 | 0 | 0.00 (0.00 - 1.05) |
| Salem | Salem Corporation | WARD 33 | 414 | 18 | 0 | 0.00 (0.00 - 0.84) |
| Salem | Salem Corporation | WARD 49 | 350 | 14 | 0 | 0.00 (0.00 - 0.97) |
| **Salem** | **Salem Corporation** | **WARD 5** | **432** | **20** | **1** | **0.24 (0.01 - 1.12)** |
| **Salem** | **Salem Corporation** | **WARD 60A** | **350** | **14** | **1** | **0.30 (0.02 - 1.40)** |
| Salem | Sankari | Chettipatti | 100 | 4 | 0 | 0.00 (0.00 - 2.66) |
| Salem | Sankari | Sanyasipatti | 50 | 2 | 0 | 0.00 (0.00 - 4.20) |
| Salem | Tharamangalam | Parakallur | 300 | 12 | 0 | 0.00 (0.00 - 1.10) |
| Salem | Veerapandy | Kalparapatti | 189 | 8 | 0 | 0.00 (0.00 - 1.64) |
| Salem | Veerapandy | Salamarathuvattam | 201 | 10 | 0 | 0.00 (0.00 - 1.60) |
| **All Sites** | | | **6898** | **304** | **2** | **0.03 (0.01 - 0.09)** |

Abbreviations: *C.q- Culex quinquefasciatus*; HUD- Health Unit Districts; 95% CI - 95% confidence interval
